# Injurious falls and subsequent adverse drug events among elderly - a Swedish population-based matched case-control study

**DOI:** 10.1186/s12877-017-0594-1

**Published:** 2017-09-04

**Authors:** C. Rausch, L. Laflamme, S. E. de Rooij, U. Bültmann, J Möller

**Affiliations:** 1 Department of Public Health Sciences, Karolinska Institutet, Widerströmska huset 4:th floor, Tomtebodavägen 18A, SE 17177 Stockholm, Sweden; 2 Department of Health Sciences, Community and Occupational Medicine, University of Groningen, University Medical Center Groningen, Antonius Deusinglaan 1, FA10, 9713 AV Groningen, The Netherlands; 3 Department of Internal Medicine, University Center for Geriatric Medicine, University of Groningen, University Medical Center Groningen, Hanzeplein 1, 9713 GZ Groningen, the Netherlands

**Keywords:** adverse drug event, poisoning, injurious falls, inappropriate drug use

## Abstract

**Background:**

Fall injuries are stressful and painful and they have a range of serious consequences for older people. While there is some clinical evidence of unintentional poisoning by medication following a severe fall injuries, population-based studies on that association are lacking. This is investigated in the current study, in which attention is also paid to different clinical conditions of the injured patients.

**Methods:**

We conducted a matched case-control study of Swedish residents 60 years and older from various Swedish population-based registers. Cases defined as adverse drug events (ADE) by unintentional poisoning leading to hospitalization or death were extracted from the National Patient Register (NPR) and the Cause of Death Register from January 2006 to December 2009 (*n* = 4418). To each case, four controls were matched by sex, age and residential area. Information on injurious falls leading to hospitalization six months prior to the date of hospital admission or death from ADE by unintentional poisoning, and corresponding date for the controls, was extracted from the NPR. Data on clinical conditions, such as dispensed medications, comorbidity and previous fall injuries were also extracted from the Swedish Prescribed Drug Register (SPDR) and NPR. Effect estimates were calculated using conditional logistic regression and presented as odds ratios (OR) and 95% confidence intervals (CI).

**Results:**

We found a three-fold increased risk of unintentional poisoning by medication in the six-month period after an injurious fall (OR 3.03; 95% CI, 2.54–3.74), with the most pronounced increase 1–3 weeks immediately after (OR, 7.66; 95% CI, 4.86–12.1). In that time window, from among those hospitalized for a fall (*n* = 92), those who sustained an unintentional poisoning (*n* = 60) tended to be in poorer health condition and receive more prescribed medications than those who did not, although this was not statistically significant. Age stratified analyses revealed a higher risk of poisoning among the younger (aged 60–79 years) than older elderly (80+ years).

**Conclusion:**

Medication-related poisoning leading to hospitalization or death can be an ADE subsequent to an episode of hospitalization for a fall-related injury. Poisoning is more likely to occur closer to the injurious event and among the younger elderly. It cannot be ruled out that some of those falls are themselves ADE and early signs of greater vulnerability among certain patients.

**Electronic supplementary material:**

The online version of this article (10.1186/s12877-017-0594-1) contains supplementary material, which is available to authorized users.

## Background

Fall injuries are a serious cause of morbidity among older people and their occurrence increases dramatically with age [1–3]. They are sustained for a variety of different environmental and individual reasons, including the health condition of older people [4–9]. Older people’s health condition may in fact imply that they are more likely to either sustain a fall or to be injured because of a fall [2, 3, 10, 11].

While medication has an important function in managing various types of health conditions, a range of studies reveal that it may increase the risk of falling among older people [8, 10, 12, 13]. This is the case for specific medications like opioids [14], certain combinations of medications and also for polypharmacy [8, 12]. The latter is preoccupying too as older people often suffer from several conditions that need to be treated simultaneously. However, even low numbers of medication used, below the polypharmacy threshold of five medications, increase the risk for falls and other injuries among older people [12, 15].

A recent clinical study revealed an association between falls and the occurrence of adverse drug events (ADE) [4]. Unintentional poisoning by medication among older people is a very severe form of ADE that can be fatal [15]. It may occur for various pathophysiological reasons [16, 17] and can be triggered by a change in medication, (e.g., in dosage, number, or type). Changes in medication, in turn, arise most typically when the health condition of older people either improves or worsens. An example of the latter is a fall-related hospitalization, which is frequent among older people [2] and has a range of documented serious short- and long-term consequences [18]. So far, however, there is no epidemiological population-based evidence on the association between injurious fall and subsequent poisoning by medication.

This study aims to determine the association between injurious falls and subsequent ADE by unintentional poisoning, and also pays attention to different clinical conditions of the fall-injured patients.

## Methods

### Study design

For the purpose of this study, a matched case-control design was nested in a national cohort of 6,981,010 individuals. Individuals born before 1959, and domiciled in Sweden at some point from 1973 onwards were identified using the Total Population Register. Information from the Swedish national health registers was linked to the cohort based on each individual’s unique personal identification number. All hospitalizations and deaths among elderly persons 60 years or older were identified for the study period of 1 January 2006 to 31 December 2009, by using the Swedish National Patient Register (NPR) and Swedish Cause of Death Register (CDR).

### Participants

Cases were defined as having been hospitalized or died due to ADE, coded as unintentional poisonings or toxic effect by medication and noxious substances using the NPR and CDR. Hospital discharge diagnoses were used (ICD-10 codes: T36–65 as main diagnosis and X40–49 as external cause of morbidity) and for mortalities (ICD-10 codes X40–49) the underlying cause of death was used. Only the first event of ADE by unintentional poisoning was considered during the study period. Cases with a main diagnosis of poisoning, but no underlying cause of poisoning related to medical treatment or medication use (ICD-10 X45-X48, including alcohol intoxication) were not considered. A total of 4418 cases were identified. Date of admission or death was used as index date for the purpose of this study.

To each case, four controls were randomly selected and matched by age (month and year), sex and residential area at the index time. Only individuals not subject to any medication-related hospitalization or death, as per case definition, during the study period were considered eligible as controls. In total 17,672 controls were matched to the cases.

### Exposure definition

Information on injurious falls leading to hospitalization 6 months prior to the index date was extracted from the NPR, and identified based on the main discharge diagnose for unintentional falls (ICD-10 code W00-W19). Only the most recent injurious falls, i.e. closest to the index date, were considered, and fall injuries with a secondary diagnosis related to sequels from prior medical care or medication used were excluded. No injurious fall during the six-month period prior to the ADE was used as reference group. Furthermore, information on previous injurious falls was extracted, and those with more than one hospitalization for a fall injury during the year prior to the index date, were categorized as repeated fallers.

### Covariates

Information on prescribed medications was extracted from the Swedish Prescribed Drug Register (SPDR), a computerized system of the pharmaceutical services, which records all the dispensations of prescribed medications at all pharmacies in Sweden through registration of the medications using the specific five-level Anatomical Therapeutic and Chemical Classification system (ATC) [19]. Exposure to medications was operationalized as number and type of prescribed and dispensed medications based on the 5th digit level of the ATC code during the four months prior to the index date. Number of prescribed medications was categorized into 0; 1, 2, 3, 4, or 1–4; 5–9; and 10 or more. One medication was considered as the reference group in the analyses. Type of medications was categorized based on medications acting on the central nervous system (CNS) (including psychotropic ATC: N05, anti-epileptic N03, psychoanaleptics N06, anti-histamine R06,, cholinesterase inhibitors N07A and anti-Parkinson medications N04), and pain relieving medication (opioid N02A and analgesics N02) was considered separately. In addition, inappropriate drug use (IDU) was assessed based on the Swedish National Board for Health and Welfare published “Indicators of appropriate drug therapy in elderly” (see Additional file 1: Appendix 1) [15].

Co-morbidity is independently associated with ADE [20, 21] and was hence considered as a confounder by using the Charlson Comorbidity Index (CCI) [22] and number of prescribed medications (as defined above). CCI was extracted from the NPR by considering the hospital discharge diagnoses according to ICD-10 codes for the three-year period prior to the index date. Seventeen different morbidities, including but not exclusive to myocardial infarction, dementia and chronic obstructive pulmonary disease, are considered in the CCI, and each category is weighted from one to six, with a level higher than five representing the most severe outcomes. CCI was categorized into four groups; 0, 1–2, 3–4 and 5 or more, with 0 serving as the reference group. The use of the CCI in population-based health register studies has been described and validated elsewhere [23, 24].

Additionally, civil status was considered as a potential confounder since previous literature has shown the protective effect of being married [25]. Civil status was extracted from the Total Population Register at time of index date and categorized into: married, unmarried, divorced, widowed or unknown. Married served as the reference group.

### Statistical analyses

Descriptive statistics were calculated to describe the characteristics of the study population with regard to demography and morbidity and a comparison was made in the distribution among cases and controls by using Pearson Chi-square tests (Table 1).

**Table 1:** Characteristics of the study population, stratified by case and control status, percentage (%), *n* = 22,090

Characteristics	Category	Percentage		*P*-value^1^
		Case *n* = 4418	Control *n* = 17,672	
Sex	Male	40.9	40.9	Matched
	Female	59.1	59.1	
Age groups (years)	60–79	51.6	51.6	Matched
	≥80	48.4	48.8	
Civil status	Married	35.5	44.3	< 0.001
	Divorced	19.5	12.8	
	Widowed	35.5	34.7	
	Unmarried	9.4	8.1	
CCI	0	56.2	81.9	< 0.001
	1–2	40.8	17.7	
	3–4	2.9	0.4	
	>4	0.1	0.0	
Number of prescribed medications	0	3.0	22.3	< 0.001
	1	1.9	8.1	
	2	2.3	8.1	
	3	3.5	8.7	
	4	4.9	8.3	
	5–9	33.0	30.0	
	>9	51.3	14.6	

^1^ *P*–value of Pearson Chi-square tests for comparison of distribution between cases and controls

Conditional logistic regression was used to assess the association between a recent fall injury and subsequent ADE by unintentional poisoning by estimating odds ratios (OR) with corresponding 95% confidence interval (95% CI). Each potential confounder was assessed in its association with the outcome and adjusted for in the main analysis when showing a statistically significant effect (*p*-value <0.05). Civil status included missing data (0.1%) that were excluded from the analysis. Analyses were performed using fall injuries in the six-month period prior to index date but were also analyzed in smaller time intervals; 1–3 months and within 1–3 months further categorized into 1–3 weeks, 4–6 weeks and 7–12 weeks were performed and presented. No injurious fall during the six-month period prior to the ADE was used as the reference group. Results are presented overall and stratified by gender and age group (60–79 years and 80+) (Table 2).

**Table 2:** Odds Ratios for ADE by time since fall injury, stratified by gender and age group, *n* = 22,090

Fall injury prior to ADE	Frequencies		OR (95% CI)		OR (95% CI) by gender and age group, adjusted			
	Cases *n* = 4418	Controls *n* = 17,672	Matched^a^	Adjusted^b^	Male^b^	Female^b^	60–79 years^b^	≥80 years^b^
No ^c^	3318	14,863	1.00 REF	1.00 REF	1.00 REF	1.00 REF	1.00 REF	1.00 REF
Yes^c^, 1–3 months	170	153	5.23 (4.17–6.58)	4.13 (3.23–5.28)	5.22 (3.44–7.94)	3.08 (2.21–4.28)	7.00 (4.27–11.5)	3.09 (2.32–4.12)
1–3 weeks	60	32	8.86 (5.76–13.6)	7.69 (4.87–12.1)	9.43 (3.56–25.0)	7.17 (4.27–12.0)	17.5 (6.23–49.6)	5.03 (2.99–8.46)
4–6 weeks	37	26	6.34 (3.83–10.5)	4.82 (2.80–8.31)	6.62 (2.80–15.6)	3.79 (1.85–7.79)	8.04 (2.92–22.1)	3.46 (1.80–6.65)
7–12 weeks	73	95	3.65 (2.76–4.99)	2.81 (2.01–3.93)	4.59 (2.71–7.77)	1.96 (1.26–3.08)	4.67 (2.37–9.06)	2.22 (1.50–3.28)
No^c^	3318	14,863	1.00 REF	1.00 REF	1.00 REF	1.00 REF	1.00 REF	1.00 REF
Yes^d^, 1–6 months	257	275	4.31 (3.62–5.15)	3.54 (2.93–4.28)	4.88 (3.53–6.76)	2.34 (1.83–2.99)	7.86 (5.33–11.6)	2.40 (1.91–3.01)

^a^Adjusted through matching by sex, age and residential area

^b^Adjusted for matching variables, civil status and CCI

^c^Individuals with a fall injury one day up to three month prior to index date

^d^Individuals with a fall injury one day up to six month prior to index date

Individuals with recent fall injuries in the time window with the highest risk for an ADE by unintentional poisoning (i.e. 1–3 weeks) were further analyzed based on their clinical characteristics before and after the hospitalization for fall injury. The distribution of characteristics is presented separately for the fall-injured with (*n* = 60) and without (*n* = 32) a subsequent ADE (Table 3). For the purpose of this, the number of medications was assessed in the four-month period prior to the fall injury. Any additional medications prescribed and dispensed between the hospitalization for a fall injury and the index date were considered as newly prescribed medications (number, specific types of medications and IDU) for the time window with the highest risk.

**Table 3:** Distribution of clinical characteristics among individuals hospitalized for fall injuries 1–3 weeks prior to the index date stratified by subsequent ADE, *n* = 92

Clinical characteristics	Category	With ADE *n* = 60% (95% CI)		Without ADE *n* = 32% (95% CI)		*p*-value^1^
Prior to the fall injury						
CCI	0	55.7	(43.1–68.3)	62.5	(45.7–79.3)	0.506
	1–2	41.7	(29.2–54.2)	35.0	(18.5–51.5)	
	3–4	3.4	(0.0–8.0)	0.0		
Number of prescribed medications^a^	0	5.0	(0.0–10.51)	9.4	(0.0–19.51)	0.367
	1–4	8.3	(1.3–15.3)	18.8	(5.2–32.3)	
	5–9	45.0	(32.4–57.6)	34.4	(17.9–50.8)	
	>9	41.7	(29.2–54.2)	37.5	(20.7–54.3)	
Repeated fallers^b^	No	73.8	(64.0–86.0)	66.7	(52.7–84.8)	0.923
	Yes	26.6	(15.5–37.9)	33.3	(17.9–50.8)	
After the fall injury^c^						
Number of new medication	0	60.0	(47.6–72.4)	66.7	(50.3–83.0)	0.525
	1–4	33.2	(21.3–45.1)	28.1	(12.5–43.7)	
	>4	6.8	(0.4–13.2)	3.1	(0.0–9.1)	
IDU	Yes	11.5	(5.0–21.8)	6.1	(1.6 – 19.6)	0.252
CNS^d^ medication (any)	Yes	28.3	(16.9–39.7)	15.6	(3.0–28.2)	0.173
Analgesics	Yes	13.3	(4.7–21.9)	3.1	(0.0–9.1)	0.155
Opioids	Yes	21.7	(11.3–32.1)	9.4	(0.0–19.5)	0.162

^1^ *P*-value of Pearson Chi-square tests for comparison of proportion between fall-injured with and without subsequent ADE

^a^Number of different medications four months prior to fall injury

^b^Considering hospitalization for any fall injury up to one year prior to index date

^c^New medications, i.e. medications the individual did not dispense in the four months prior to the fall injury. The presented medication categories are not mutually exclusive

^d^Any medication acting on the central nervous system (CNS)

IBM SPSS Statistics 22 was used to perform the statistical analyses.

### Ethical approval

According to Swedish regulations, the Personal Data Act (Personuppgiftslagen) § 10 [26], the ethical application specified why the researchers did not consider informed consent as necessary – or feasible (as in the case of a large register-based study such as ours). The study was approved by the Regional Ethical Review Committee in Stockholm, Sweden (2010/865–31/2 and 2011/15–32).

## Results

Table 1 shows that the majority of cases were female (59.1%), were 60–79 years old (51.6%). In comparison to the controls, cases were more likely to be subject to co-morbidity (CCI >0: 18.1 vs. 43.8%) and had more often prescribed medications (77.7 vs. 97.0%). Among the cases, more than 50% had ten or more medications, compared to only approximately 15% among the controls. Among the controls, 22% did not have any prescribed medications whereas the corresponding number among cases was only 3%.

Table 2 shows the odds ratios for ADE by unintentional poisoning in different time windows since injurious fall, stratified by gender and age group. After taking confounding from civil status and comorbidity into account, the odds of an ADE is higher among elderly with an injurious fall during the last six months (OR, 3.54; 95% CI, 2.93–4.28) compared to those without. The most pronounced increased risk was in the 1–3 weeks after the fall injury (OR 7.69; 95% CI, 4.87–12.1), and decreased over the six-month period but remained statistically significant during the whole six-month period. Older people aged 60–79 years showed the highest risk, specifically in the 1–3 week time window. No gender differences were seen, except when considering the ADE within the six months after the injurious fall (male OR, 4.88; 95% CI, 3.53–6.76; female OR, 2.34; 95% CI, 1.83–2.99).

In Table 3, the clinical characteristics of older people with an injurious fall, with and without a subsequent ADE in the 1–3 weeks, are presented in order to underline the potential differences with regard to prescribed medications and comorbidity before and after the fall injury. Type of fall injury (according to external cause of fall injury) did not differ between the two groups and neither did length of stay in the hospital due to the fall injury (for those with a subsequent ADE) on average 9.4 days (SD 9.1) and for those without a subsequent ADE 9.5 days (SD 8.5) (results not shown), indicating similarly serious fall injuries in both groups. Although not statistically significant, individuals with a subsequent ADE after an injurious fall tend to have a higher comorbidity score (CCI >0: 44.3 vs. 35.0%), but tend to be less likely to be repeated fallers (26.6 vs. 33.3%). Further, they seem to be more likely to have received opioids (21.7 vs. 9.4%) and analgesics (13.3 vs. 3.1%) The most commonly newly prescribed pharmaceutical drug groups were opioids (N02A), other analgesics and antipyretics (N02B), with paracetamol (*n* = 11) and oxycodone (*n* = 7) as the most commonly newly prescribed medications.

## Discussion

In this large population-based study, we found that the risk of unintentional poisoning by medication is tripled in the six-month period following an episode of hospitalization for an injurious fall, with the excess risk peeking 1–3 weeks immediately after the fall. This echoes findings from an earlier clinical study [4]. Previous studies have also considered the role of co-morbidity, polypharmacy and social factors, i.e. civil status in regard to ADE [20, 25, 27] and found similar results. While these studies used broader definitions of ADE or focused predominantly on the clinical setting, our population-based study focused on a more severe ADE, defined by hospitalized and fatal unintentional poisoning events. In our data, the association remained after adjustment for the patients’ clinical condition (assessed herein by CCI) and civil status. In the peak period, among the fallers, those who had a subsequent poisoning tended to be in poorer health condition and receive more prescribed medications than those who were not, but were also less likely to have a previous history of fall-related hospitalizations. Compared to other studies concerned with length of hospital stay as risk factor for ADE [28, 29], we did not find any significant difference between length of hospital stay for the injurious falls with and without ADE in the peak period. The association between injurious fall and ADE differs by age, where age-stratified analyses revealed a higher risk among the younger (aged 60–79 years) than older elder (80+ years).

As mentioned earlier, medication has the positive benefit of helping to manage various types of health conditions among older people. But in some circumstances they may have caused adverse effects, among which fall and injurious falls are well documented ones [8, 10, 12, 13] and unintentional poisoning, less so [15]. What makes severe injurious falls and subsequent unintentional poisoning related to one another may have different explanations. One being that the clinical condition of some of those who sustain severe fall injuries is poor and that the fall itself is more or less a reflection of their vulnerability to ADEs [4, 6]. In that case, suffering from unintentional poisoning is an additional manifestation of this vulnerability. In our study, however, we only found an indication of such preconditions that would differentiate poisoned and non-poisoned fallers.

An alternative explanation is that sustaining an injurious fall implies changes or adjustments in the patient’s drug treatment (e.g., change in dosage, number, and types of medications) and that this in itself may increase the risk of ADE. That could explain why, in this study, the excess risk of poisoning is so much more pronounced shortly after the fall when changes in drug treatment are more likely to occur. Clinical studies have shown that changes in medication regiment can increase the risk for ADE [20, 30].

Physicians might be less inclined to consider atypical presentation of diseases or geriatric syndromes among younger elder [6] and focus their intervention on the fall injury itself rather than on potential external causes like medication use or co-morbidity. This is less likely in the case of the older elder as there are guidelines for these situations [20].

To our knowledge, this study is one of the few in the area and the first population-based one assessing the association between injurious falls and subsequent unintentional poisoning; with only one previous study in a clinical setting and with smaller sample size (*n* < 1000) [4]. One major strength is the linkage of individual information accessed from Swedish registers which significantly increases the size of the study (nationwide), with high coverage and quality of the assessment of ADE, injurious falls and potential covariates [31]. Using register data eliminates the risk of recall bias, which is often a problem in case-control studies, though we can only generalize our findings to fall injuries and poisonings resulting in hospitalization.

Information concerning medication over the counter was not accessible for the study. Although this may lead to important interactions being unnoticed, it applies equally to cases or controls. It is also of note that we used four months prior to the index date as a reference period. This has the advantage of capturing chronic medication use with long prescription routines (in Sweden, medications are normally prescribed for a treatment period of three months) but it may mean that we miss medications prescribed on a needs basis. This could be important especially among older people with an injurious fall, who often are advised to take pain-relieving medication when needed.

Identification of unintentional poisoning and toxic effects by medication and noxious substances (regarded herein as an ADE) was based on specific and appropriate ICD diagnoses from the hospitalization and death register and this approach has also been employed in previous studies with other risk factors [29, 32]. However, we cannot rule out some degree of inaccuracy in reporting to the registers as physicians might misdiagnose ADE with unspecific clinical presentations, especially among older individuals [33–35].

As in previous studies, we adjusted for co-morbidity and social factors, i.e. civil status [20, 25, 27]. We assessed comorbidity using the Charlson Comorbidity Index which has been described and validated for register-based studies elsewhere [23, 24]. We acknowledge, though, that there might be some residual confounding so as an alternative measure of comorbidity, we also adjusted for number of prescribed medications prior to the index date. Such adjustment did not further reduce the effect. Having adjusted for CCI, we refrained from including another proxy for frailty to avoid over-adjustment and as medications prescribed after the fall injury might act as mediators. However, we cannot rule out that even minor changes in health conditions, such as acute infections that are not captured in registers, could contribute to the vulnerability of poisoning among fall injured.

## Conclusions

This study shows epidemiological evidence that medication-related poisoning leading to hospitalization or death can be an adverse drug event subsequent to an episode of hospitalization for a fall-related injury. Poisoning is more likely to occur closer to the injurious event and among older elder. It cannot be ruled out that some of those falls are themselves ADE and early signs of greater vulnerability among certain patients.

## Additional file

Additional file 1: Appendix 1. 15–08-2017. Description of the variable inappropriate drug use. File contains information on the drugs that were considered for the specific groups of “inappropriate drug use”. (DOCX 17 kb)

## Abbreviations

ADE: adverse drug events
ATC: Anatomical Therapeutic and Chemical Classification system
CCI: Charlson Comorbidity Index
CDR: Swedish Cause of Death Register
CNS: central nervous system
IDU: inappropriate drug use
NPR: National Patient Register
SPDR: Swedish Prescribed Drug Register
