# Supplementary material for: Injurious falls and subsequent adverse drug events among elderly - a Swedish population-based matched case-control study
Source: BMC Geriatr. 2017 Sep 4;17:202. doi: 10.1186/s12877-017-0594-1 (PMC5583997; doi:10.1186/s12877-017-0594-1)
Supplement: Additional file 1: — Appendix 1. 15–08-2017. Description of the variable inappropriate drug use. File contains information on the drugs that were considered for the specific groups of “inappropriate drug use”. (DOCX 17 kb) [file 12877_2017_594_MOESM1_ESM.docx]

# Appendix 1. (description of the variable inappropriate drug use)

The following criteria were used to assess inappropriate drug use, based on the Swedish Board for Health and Welfare [33].

**Single drug criteria**

Except in specific circumstances these medications should be avoided among elderly [33].

- dispensation of long-acting benzodiazepines
- dispensation of anti-cholinergic medication
- dispensation of Tramadol
- dispensation of Propiomazin

**Multiple medication criteria**

- excessive polypharmacy (10 or more medications dispensed) (is accounted for in variable number of prescribed medications)
- coinciding treatment with two medications from similar ATC groups
- coinciding treatment with three or more psychotropic medications

**Drug-Drug interaction criteria**

Includes the most commonly identified drug-drug interactions in Sweden, according to Holm *et al.*[34]:

- quinolone/tetracycline-metal ion,
- potassium-potassium-sparing diuretics,
- warfarin-acetylsalicylic acid,
- proton pump inhibitor-clopidogrel,
- calcium antagonist-beta blocker,
- tramadol-antidepressant,
- codeine/ethylmorphine-antidepressant,
- warfarin-NSAID,
- verapamil-digoxin,
- diazepam-carbamazepine,
- calcium antagonist-carbamazepine,
- SSRI-SSRI,
- warfarin-antimycotic,
- risperidone/quetiapine-carbamazepine and
- dopamine antagonist-dopamine agonist
